# Society Meetings

**Published:** 1913-04

**Authors:** 


### SOCIETY MEETINGS.

The American Association of Orificial Surgeons will hold its spring clinic at Hering Medical College, Wood and York streets, Chicago, April 23-26.

---

I. C. I. Chapter of Nu Sigma Nu held an open meeting, to which the alumni were invited, at the Chapter House, 246 Elmwood Ave., Buffalo, March 3.

---

Medical Society of the State of New York. At the next meeting of the Medical Society of the State of New York, in Rochester, April 28 to May 1, it is planned to have an exhibit of scientific interest. Doctors in this vicinity are requested to loan exhibits of this nature. Each exhibit should be marked so that those viewing it would know whence it came, and the school and year of graduation of the practitioner. Communications may be addressed to Dr. Wesley T. Mulligan, Rochester, N. Y.

---

At the regular meeting of the Medical Union of Buffalo, held at the Statler Hotel, March 27, Dr. George Edward Fell gave an illustrated paper on Ethmoiditis. Transverse and longitudinal sections of the skull were made and protographed by the author for projection. The method of operation known as the "Moshier" was illustrated and described in an interesting manner."

The regular meeting of the Medical Society of the County of Monroe was held at the rooms of the Rochester Academy of Medicine March 18. Subject of paper, "Intestinal and Lymphatic Chyle, With Special Reference to Clinical Aspects," Dr. A. L. Benedict, Buffalo.

Dr. Young reported that, with special reference to the meeting of the State Society, April 29-May 1, a circular letter had been sent to physicians west of the eastern line of Oneida County, urging them to become members of the State and County organizations. One thousand five hundred and twenty-three unaffiliated physicians were listed for this territory, the special field of this journal, 384 of whom were designated as homoeopaths or eclectics.

Memorial resolutions were adopted regarding Drs. Frank A. Jones and A. W. Bellam, recently deceased.

The Medical and Surgical League held its monthly meeting March 13. Dr. Robert Mehnert read a paper entitled, "Asthma." Dr. Alfred Noehren reported a case of sarcoma of the testicle upon which he had operated, but in which a metastasis later formed in the spinal canal giving rise to interesting symptoms.

The Utica Medical Library Association is endeavoring to awaken renewed interest among its members by securing papers from men of prominence outside the society. The paper of the February meeting was a very excellent address on Cardiac Irregularities by Dr. W. D. Alsever of Syracuse, and was heard with much interest. After the paper and its discussion an Italian luncheon was served. At the March meeting the literary program was followed by a Mexican luncheon.

The promise of Hon. P. C. J. DeAngelis, Justice of Supreme Court, has been secured to deliver a paper before this Association at its April meeting on "The Medical Expert," or "Expert Testimony."

